# Supplementary material for: SO2 and copper tolerance exhibit an evolutionary trade-off in Saccharomyces cerevisiae
Source: PLoS Genet. 2023 Mar 28;19(3):e1010692. doi: 10.1371/journal.pgen.1010692 (PMC10081759; doi:10.1371/journal.pgen.1010692)
Supplement: S1 Text — Fig A: The mean difference in growth of spores in defined medium containing 0.25 mg/L relative to medium containing 10 mg/L of copper. Spores were isolated from yeast strains AWRI 3001 and AWRI 3811. Growth of spores was assessed in microplates and 100 mL cultures for AWRI 3001 and AWRI 3807 respectively. Yeast growth was assessed as absorbance at 600 nm after 72 h incubation at 17°C. Bars show the mean difference in absorbance (600 nm) with error bars indicating the 95% confidence interval (n = 2). Bars are coloured by the pool to which each spore was assigned for bulk segregant analysis. Fig B: Comparison of haploid CUP1 copy number variation (CNV) as estimated by Steenwyk et al [15] and absolute CNV estimated by Onetto (this work) in yeast strains common to both studies. CNV estimated by Onetto et al are the mean of at least three independent estimates with error bars showing standard deviation. Fig C: Growth of spores isolated from yeast strains A) 3019, B) 3029 and C) 3032 in defined medium containing 0.25 mg/L (blue) or 10 mg/L (red) of copper. Yeast growth was assessed as absorbance at 600 nm after 48 h (A and C) or 72 h (B) incubation at 17°C. Error bars show the mean of 3 (B) or 4 (A and C) replicates. Growth of the diploid parent for each set in both conditions is also shown (parent). Fig D: Over-representation analysis of transcripts with differential abundance in an SSU1 over-expressing strain. Transcript abundance in AWRI 4052 was compared to transcript abundance in the cognate unmodified strain AWRI 3471 growing in defined medium containing 10 mg/L copper. Transcript classes that were over-represented in the SSU1 over-expressing strain are shown. Fig E: Over-representation analysis of proteins with differential abundance in an SSU1 over-expressing strain. Protein abundance in AWRI 4052 was compared to protein abundance in the cognate unmodified strain AWRI 3471 growing in defined medium containing 10 mg/L copper. Protein classes that were over-represen [file pgen.1010692.s001.docx]

# Supplementary Material


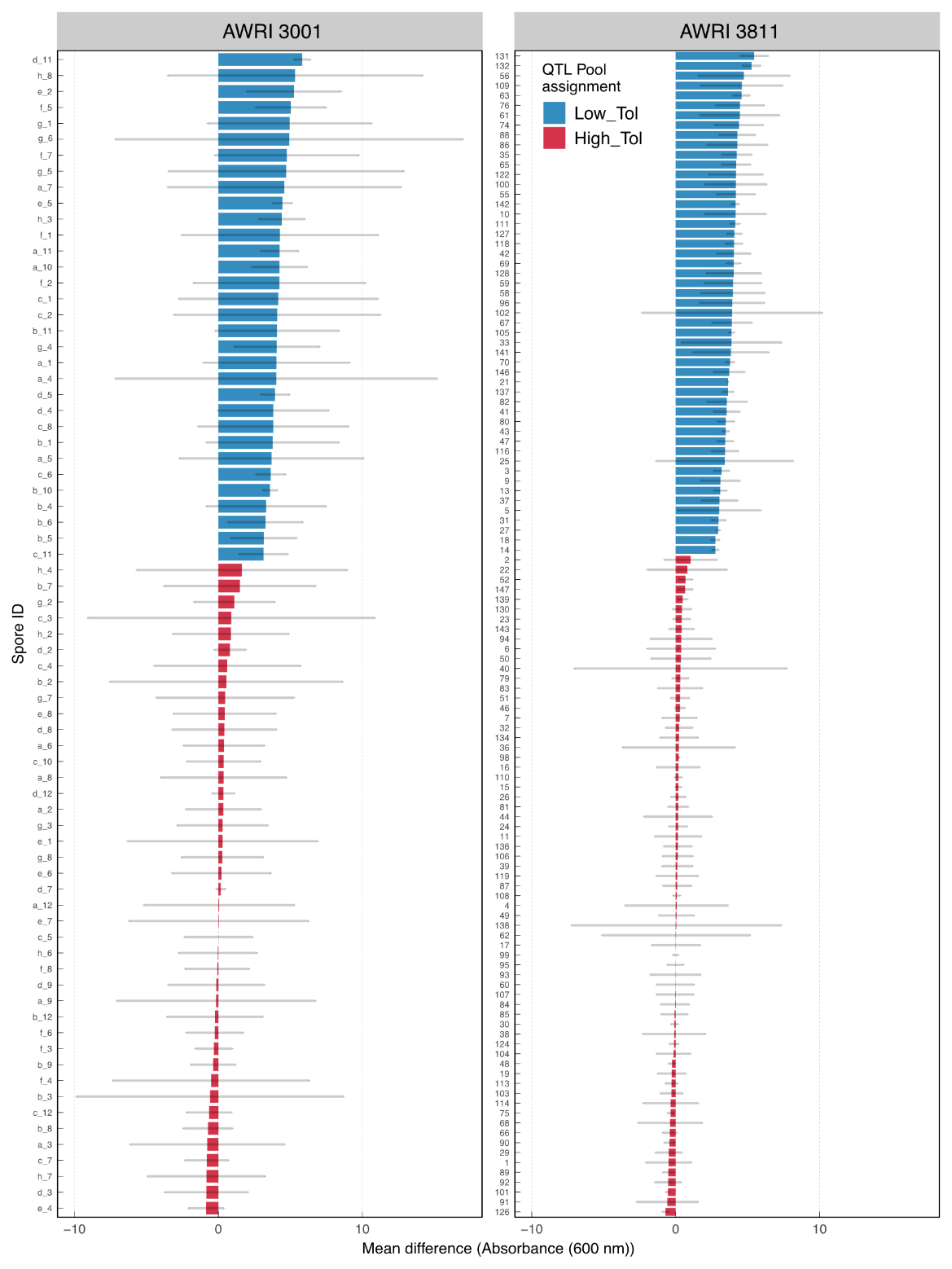


Fig A. The mean difference in growth of spores in defined medium containing 0.25 mg/L relative to medium containing 10 mg/L of copper.

Spores were isolated from yeast strains AWRI 3001 and AWRI 3811. Growth of spores was assessed in microplates and 100 mL cultures for AWRI 3001 and AWRI 3807 respectively. Yeast growth was assessed as absorbance at 600 nm after 72 h incubation at 17 °C. Bars show the mean difference in absorbance (600 nm) with error bars indicating the 95% confidence interval (n = 2). Bars are coloured by the pool to which each spore was assigned for bulk segregant analysis.


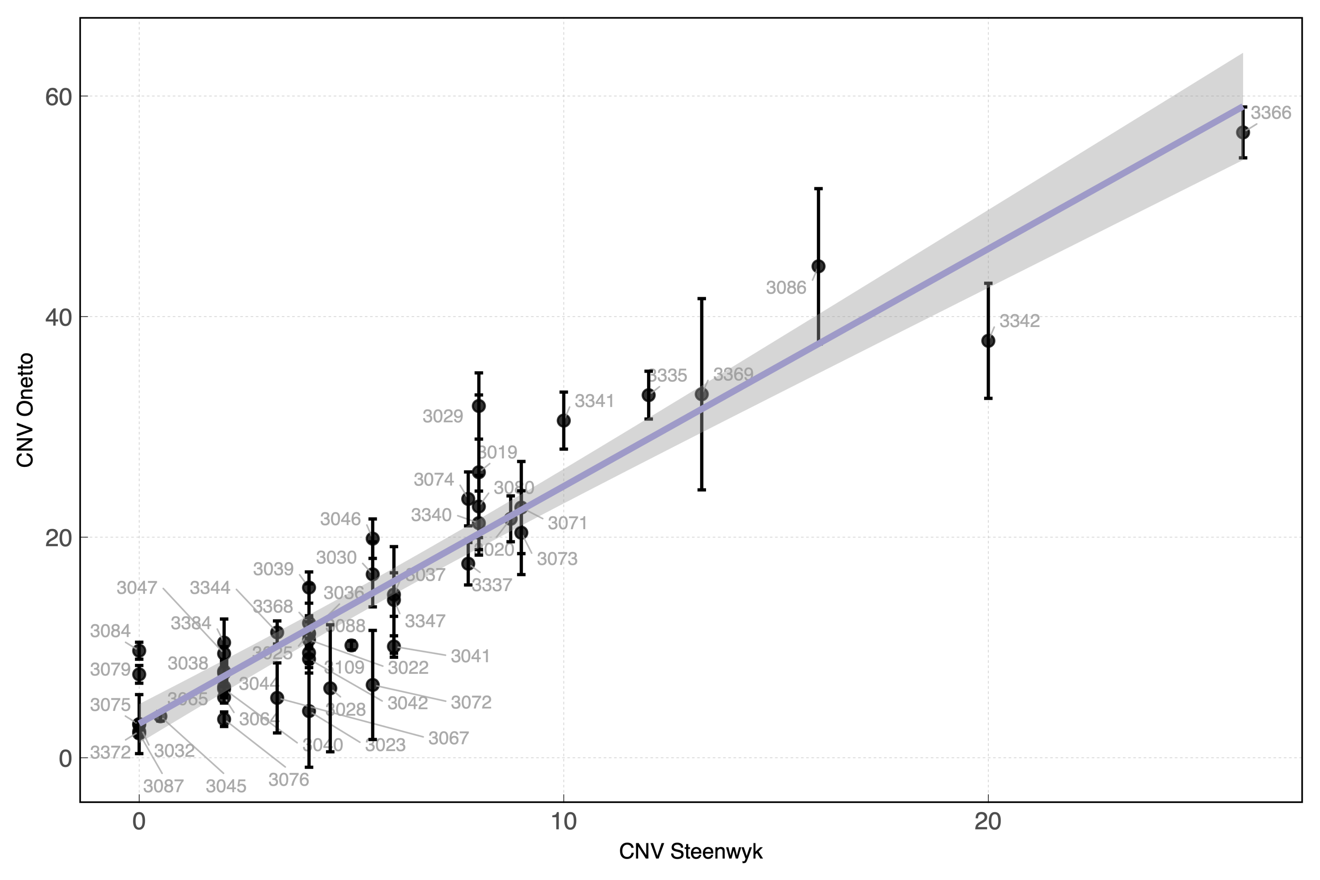


Fig B. Comparison of haploid CUP1 copy number variation (CNV) as estimated by Steenwyk et al [1] and absolute CNV estimated by Onetto (this work) in yeast strains common to both studies.

CNV estimated by Onetto et al. are the mean of at least three independent estimates with error bars showing standard deviation.


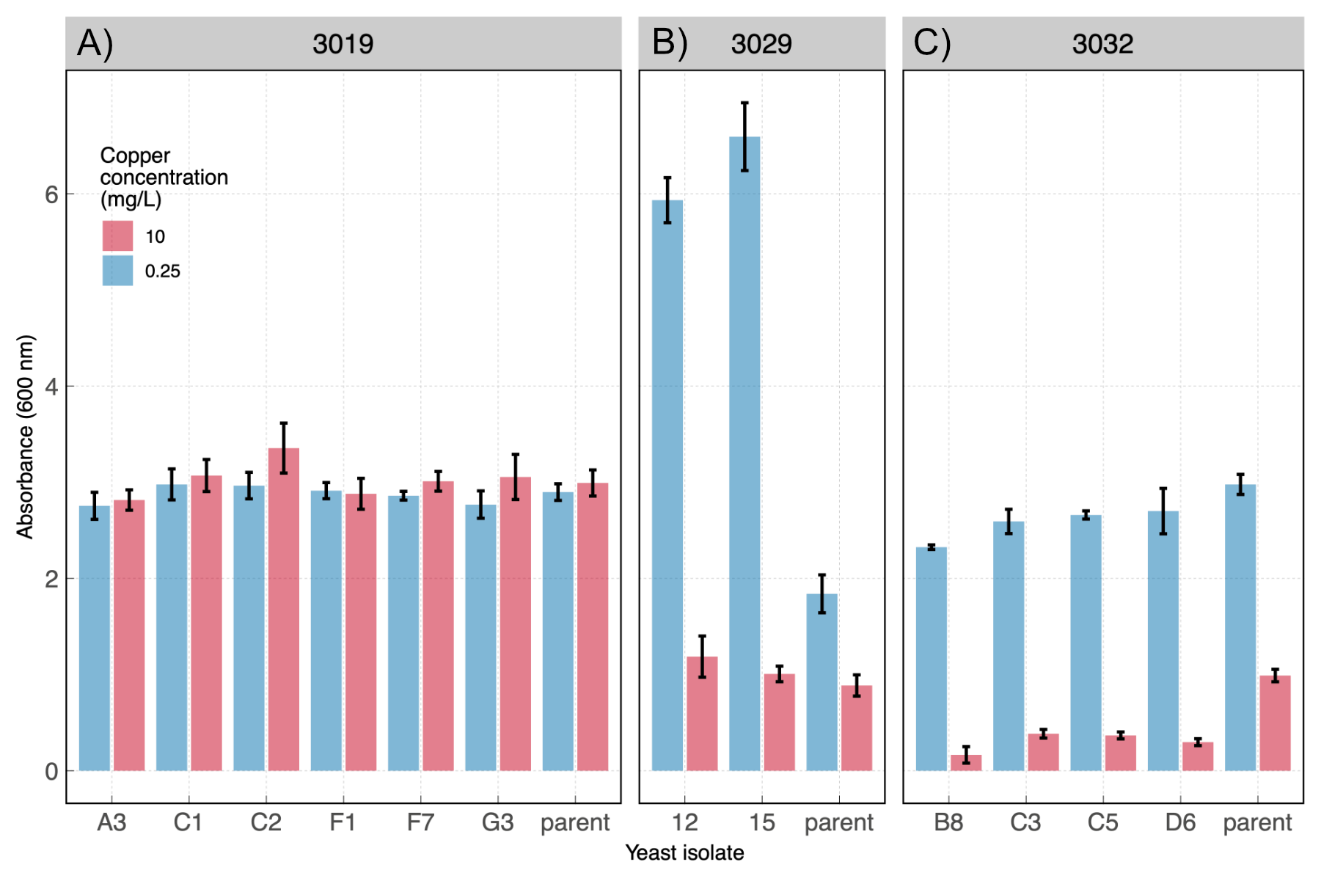


Fig C. Growth of spores isolated from yeast strains A) 3019, B) 3029 and C) 3032 in defined medium containing 0.25 mg/L (blue) or 10 mg/L (red) of copper.

Yeast growth was assessed as absorbance at 600 nm after 48 h (A and C) or 72 h (B) incubation at 17 °C. Error bars show the mean of 3 (B) or 4 (A and C) replicates. Growth of the diploid parent for each set in both conditions is also shown (parent).


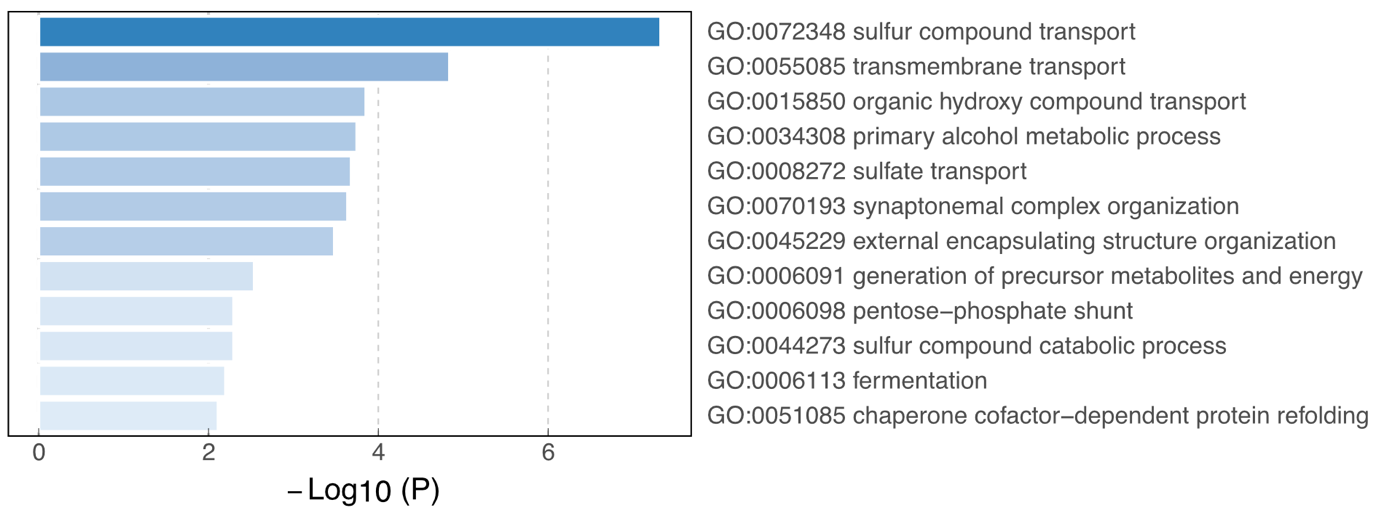


Fig D. Over-representation analysis of transcripts with differential abundance in an *SSU1* over-expressing strain.

Transcript abundance in AWRI 4052 was compared to transcript abundance in the cognate unmodified strain AWRI 3471 growing in defined medium containing 10 mg/L copper. Transcript classes that were over-represented in the *SSU1* over-expressing strain are shown.


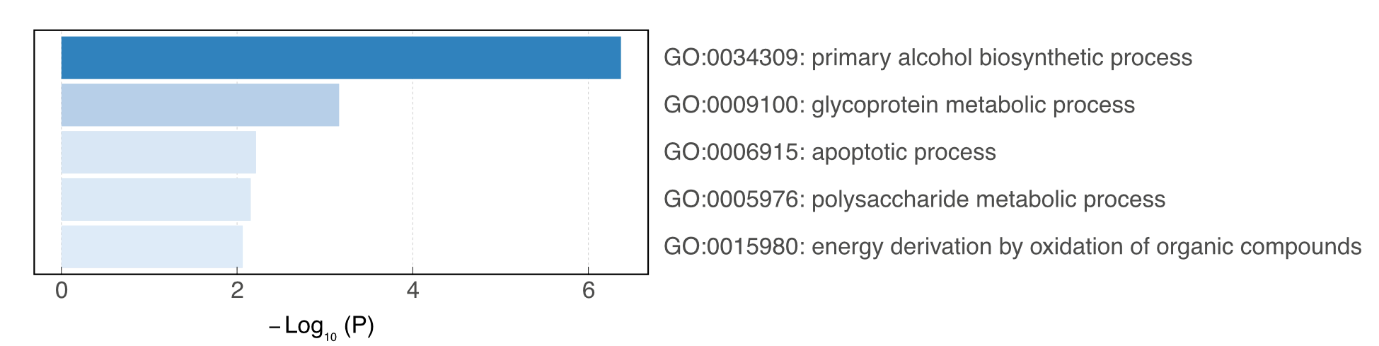


Fig E. Over-representation analysis of proteins with differential abundance in an *SSU1* over-expressing strain.

Protein abundance in AWRI 4052 was compared to protein abundance in the cognate unmodified strain AWRI 3471 growing in defined medium containing 10 mg/L copper. Protein classes that were over-represented in the *SSU1* over-expressing strain are shown.


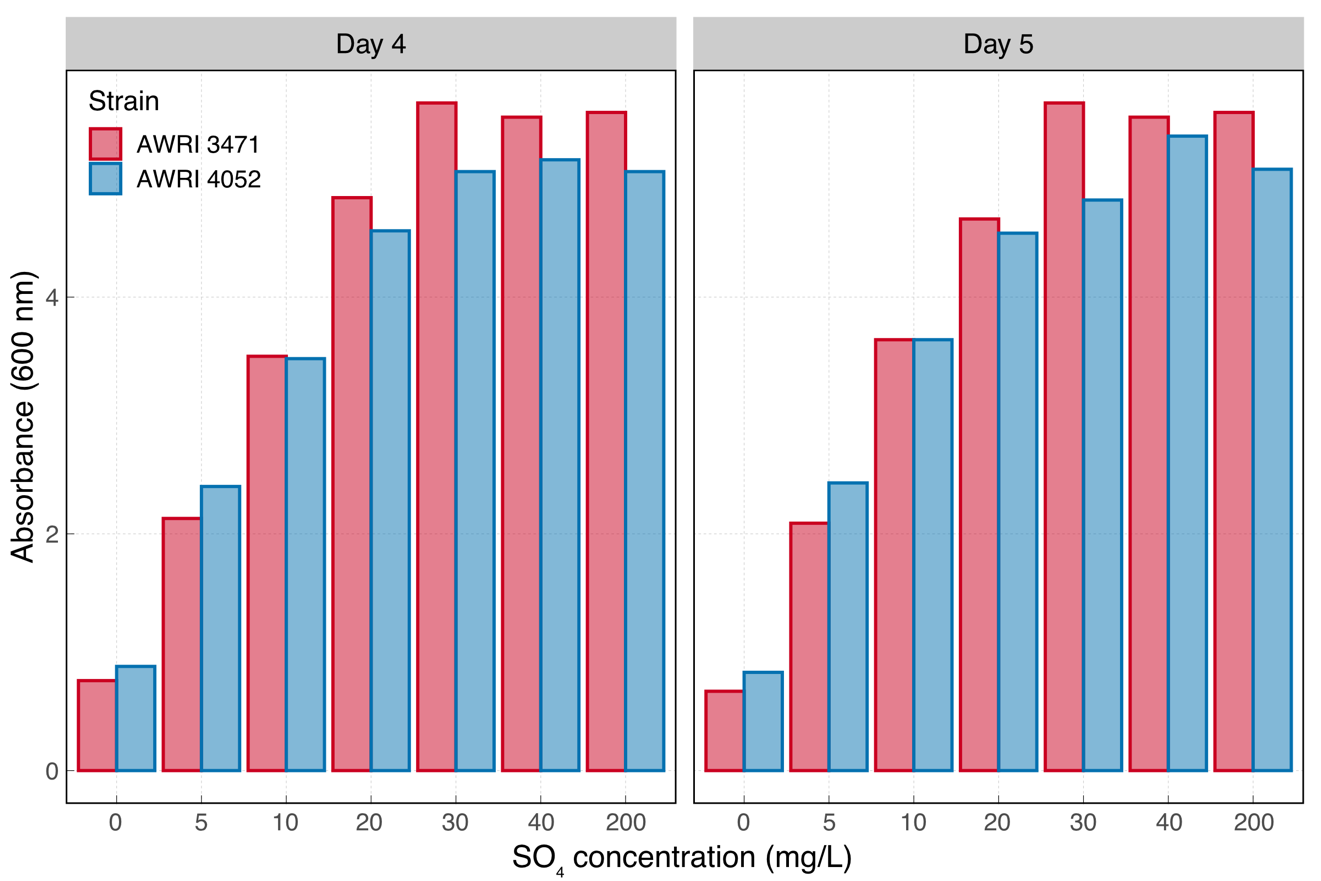


Fig F. Effect of sulfate concentration on the growth of yeast strains AWRI 3471 (red) and AWRI 4052 (blue).

Growth was determined by measuring absorbance at 600 nm. Yeast strains were grown in defined medium using 100 mL fermentation vessels. The height of each bar is the absorbance reading for a sample (n=1). Data collected on day 4 and day 5 are shown. This information was used to estimate a suitable concentration of sulfate to use in subsequent experimental work.


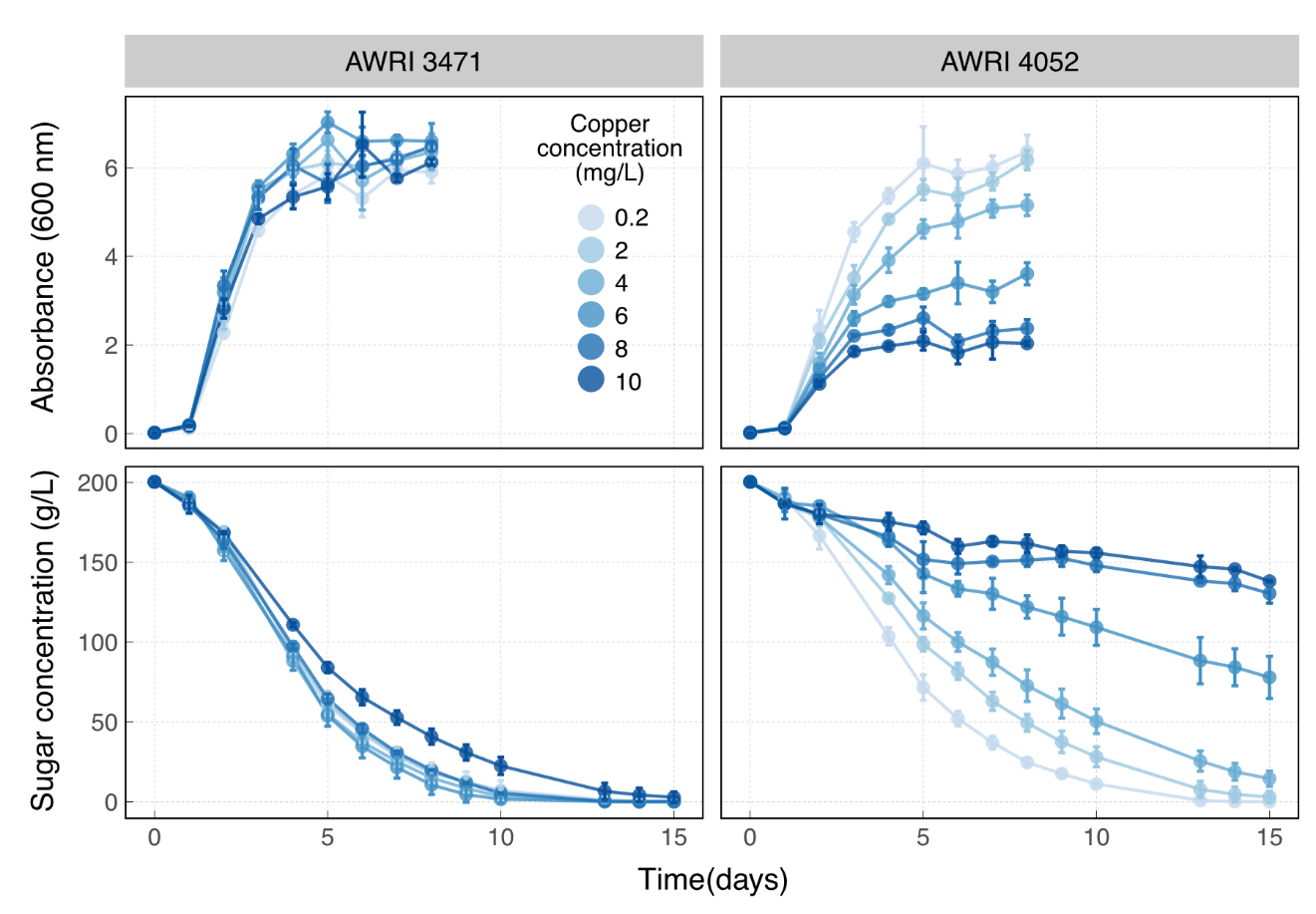


Fig G. The effect of increasing copper concentrations on the growth and fermentation kinetics of yeast strains AWRI 3471 and AWRI 4052

Yeast were grown in defined medium containing an estimated 20 mg/L SO_4_. Growth was followed by measuring absorbance at 600 nm. Sugar concentration is the sum of glucose and fructose concentrations measured enzymatically. Points show means (n = 3) and error bars show standard deviation.

# Supplementary Tables

Table A. Primers used in this work. Primers used for chromosomal translocation status are taken from Zimmer et al. [2].

| Primer name | Primer sequence |  |
| --- | --- | --- |
| Illumseq1 | cctacacgacgctcttccgatct |  |
| Illumseq2 | cagacgtgtgctcttccgatct |  |
| barcode_F | cctacacgacgctcttccgatct |  |
| barcode_R | cagacgtgtgctcttccgatct |  |
| CUP1F_SS | ccaaaatgaaggtcatgagtgccaat |  |
| CUP1R_SS | gagcagcatgacttcttggtttcttc |  |
| Primer_MAT | agtcacatcaagatcgtttatgg |  |
| Primer_MATa | actccacttcaagtaagagtttg |  |
| Primer_MATalpha | gcacggaatatgggactacttcg |  |
| CORE1_Ampl-F | gggatagcgtcaagatgacacttctacttttttgtgacacatcatcatgcatccccgggttaattaaggc |  |
| CORE1_Ampl-R | atgaaggggtcaaactgcctcgtaagagcaagtacccaattggcaaccatactatagggagaccggcaga |  |
| Chr16_SSU1_Prom-F | aaaccgatttaaattatccaa |  |
| Chr16_SSU1_Prom-R | gtagtgcctgcacagcaatg |  |
| p416hy-FR1-F | aaaggatctaggtgaagatcct |  |
| FR2-URA3-p416-R | ccgacaggactataaagatacca |  |
| P758 | aaagaagttgcatgcgccta |  |
| P762 | acctatcgagtctcccac |  |
| P763 | ccatatttgtgatgatatcg |  |
| P764 | tcgaacatcgagcatgca |  |
| P765 | gacacccatgaccatcac |  |
| P786 | cgcatccagtacaaagaaatg |  |
| P787 | ctgagtgatttgtttcccga |  |
| P788 | tctttttgggctggtaggat |  |
| P789 | atatttgtagtgcctgcaca |  |
| pChrXVwt | tgctggatgagaaacgagtg |  |

Table B. Primer pairs used to detect chromosomal rearrangements.

| Pair | Chromosomal arrangement detected | Observed fragment size range (bp) |
| --- | --- | --- |
| P788/789 | Chr XVI wild type | 1080-1326 |
| P762/p789 | Chr XVI wild type | 843-932 |
| P786/p787 | Chr VIII wild type | 1029-1323 |
| P762/p763 | Chr XVI :: Chr VIII translocation | 496-514 |
| P764/p789 | Chr VIII :: Chr XVI translocation | 572-1055 |
| P758/pChrXVwt | Chr XV wild type | 2514-2279 |
| P765/p758 | Chr XV :: Chr XVI translocation | 756-797 |

Table C. Effect of Copper concentration and plasmid containing *MET 3/13/16* genes on SO_2_ production by AWRI 3471.

SO_2_ concentration is given as the mean if three replicates (Mean SO_2_) with standard deviation (sd) shown. Two-way ANOVA analysis of was conducted with yeast strain, copper concentration and yeast * copper investigated as factors at alpha = 0.05. The table shown gives the results of the ANOVA and a TUKEY multiple pairwise comparison evaluating the magnitude of differences between pairs of treatments.

| **Treatment** | **df** | **sum sq** | **mean Sq** | **F stat** | **P** |
| --- | --- | --- | --- | --- | --- |
| strain | 1 | 184.9 | 184.9 | 143.7 | 2.16E-06 |
| Cu_conc | 1 | 42.9 | 42.9 | 33.4 | 4.15E-04 |
| strain:Cu_conc | 1 | 70.5 | 70.5 | 54.8 | 7.60E-05 |
| Residuals | 8 | 10.3 | 1.3 | *NA* | *NA* |
|  |  |  |  |  |  |
| **Strain** | **Copper concentration** | **Mean SO_2_**  **(mg/L)** | **sd** |  |  |
| 3471 [NatR] | Hcu | 4.1 | 0.8 |  |  |
| 3471 [NatR] | Lcu | 3.0 | 1.1 |  |  |
| 3471 [NatR, MET+] | Hcu | 7.1 | 1.0 |  |  |
| 3471 [NatR, MET+] | Lcu | 15.7 | 1.5 |  |  |
|  |  |  |  |  |  |
| **Contrast** | ***Mean Difference*** | ***P*** | ***Lower CI*** | ***Upper CI*** |  |
| 3471 [NatR]:Hcu - 3471 [NatR]:Lcu | 1.1 | 6.73E-01 | -1.9 | 4.0 |  |
| 3471 [NatR]:Hcu - 3471 [NatR, MET+]:Hcu | -3.0 | 4.72E-02 | -6.0 | 0.0 |  |
| 3471 [NatR]:Hcu - 3471 [NatR, MET+]:Lcu | -11.6 | 0.00E+00 | -14.6 | -8.7 |  |
| 3471 [NatR]:Lcu - 3471 [NatR, MET+]:Hcu | -4.1 | 9.90E-03 | -7.0 | -1.1 |  |
| 3471 [NatR]:Lcu - 3471 [NatR, MET+]:Lcu | -12.7 | 0.00E+00 | -15.7 | -9.7 |  |
| 3471 [NatR, MET+]:Hcu - 3471 [NatR, MET+]:Lcu | -8.6 | 1.00E-04 | -11.6 | -5.7 |  |

Table D. Effect of Copper concentration and plasmid containing *MET 3/13/16* genes on SO_2_ production by AWRI 4052.

SO_2_ concentration is given as the mean if three replicates (Mean SO_2_) with standard deviation (sd) shown. Two-way ANOVA analysis was conducted with yeast strain, copper concentration and yeast * copper investigated as factors at alpha = 0.05. The table shows the results of the ANOVA and TUKEY multiple pairwise comparison evaluating the magnitude of differences between pairs of treatments.

| **treatment** | **df** | **sum sq** | **mean Sq** | **F stat** | **P** |
| --- | --- | --- | --- | --- | --- |
| strain | 1 | 1.2 | 1.2 | 0.1 | 8.15E-01 |
| Cu_conc | 1 | 3.5 | 3.5 | 0.2 | 6.87E-01 |
| strain:Cu_conc | 1 | 479.6 | 479.6 | 23.6 | 1.26E-03 |
| Residuals | 8 | 162.4 | 20.3 | NA | NA |
|  |  |  |  |  |  |
| **strain** | **Copper concentration** | **Mean SO_2_**  **(mg/L)** | **sd** |  |  |
| 4052 [NatR] | Hcu | 43.3 | 4.2 |  |  |
| 4052 [NatR] | Lcu | 29.5 | 2.7 |  |  |
| 4052 [NatR, MET+] | Hcu | 30.0 | 3.9 |  |  |
| 4052 [NatR, MET+] | Lcu | 41.5 | 6.4 |  |  |
|  |  |  |  |  |  |
| **Contrast** | ***Mean Difference*** | ***P*** | ***Lower CI*** | ***Upper CI*** |  |
| 4052 [NatR]:Hcu – 4052 [NatR]:Lcu | 13.7 | 2.39E-02 | 1.9 | 25.5 |  |
| 4052 [NatR]:Hcu - 4052 [NatR, MET+]:Hcu | 13.3 | 2.84E-02 | 1.5 | 25.1 |  |
| 4052 [NatR]:Hcu - 4052 [NatR, MET+]:Lcu | 1.7 | 9.64E-01 | -10.1 | 13.5 |  |
| 4052 [NatR]:Lcu - 4052 [NatR, MET+]:Hcu | -0.5 | 9.99E-01 | -12.2 | 11.3 |  |
| 4052 [NatR]:Lcu - 4052 [NatR, MET+]:Lcu | -12.0 | 4.58E-02 | -23.8 | -0.2 |  |
| 4052 [NatR, MET+]:Hcu - 4052 [NatR, MET+]:Lcu | -11.6 | 5.45E-02 | -23.3 | 0.2 |  |

# References

1. Steenwyk J, Rokas A. Extensive Copy Number Variation in Fermentation-Related Genes Among Saccharomyces cerevisiae Wine Strains. G3; Genes|Genomes|Genetics. 2017;7: 1475–1485. doi:10.1534/g3.117.040105

2. Zimmer A, Durand C, Loira N, Durrens P, Sherman DJ, Marullo P. QTL dissection of Lag phase in wine fermentation reveals a new translocation responsible for Saccharomyces cerevisiae adaptation to sulfite. Schacherer J, editor. Plos One. 2014;9: e86298. doi:10.1371/journal.pone.0086298
