# Supplementary material for: SO2 and copper tolerance exhibit an evolutionary trade-off in Saccharomyces cerevisiae
Source: PLoS Genet. 2023 Mar 28;19(3):e1010692. doi: 10.1371/journal.pgen.1010692 (PMC10081759; doi:10.1371/journal.pgen.1010692)
Supplement: Attachment — Submitted filename: 20230104_ResponseToReviewersComments.docx [file pgen.1010692.s002.docx]

Dear editor and reviewers,

The authors would like to thank you for your time in reviewing this manuscript. The commentary by the reviewers resulted in us looking more closely at how the work was presented. In addition to the changes suggested by the reviewers we have re-written some sections to improve comprehension. All changes have been recorded using tracked changes in word except for changes to the references and in-text citations which would have distracted from the more substantive responses to reviewers. We believe that this work has been improved and is easier to interpret as a result of the suggestions provided by all. We hope that the work is now suitable for publication in Plos Genetics. Point-by-point responses to reviewer comments are provided below.

Editor comment: All three reviewers were positive but brought up important issues that would improve the manuscript. I would like to emphasize one point. The relationship between CUP1 copy number and resistance should be clarified in relation to prior work. Is this due to the copper concentration, the medium to which copper was added or some other factor. The significance of your findings is related to a careful explanation of how copper resistance was measured. Rather than citing reference 7, please include the medium and copper concentration, and the formulation (since g/mL differs depending on the form of copper used). A reasonable but unanswered question that a reader might have is whether SSU1 genotype affects copper resistance in rich or complete medium - probably the most commonly used assays.

Author response:

It was not our intention to give the impression that *CUP1* does not contribute to copper tolerance in wine yeast. In fact, our QTL analysis supports the role of *CUP1* in copper tolerant phenotypes whether that is in YPD or wine-like defined media. To clarify this point we have added some text to the QTL section where we state that;

“This data explains the copper sensitivity of the AWRI 1537 strain and supports previous observations [18,19] that *CUP1* is a key determinant of copper tolerance in yeast.”

We have also explicitly outlined our medium composition and provided the form in which copper was added. We did not intend to omit these details from the original submission.

The question about whether *SSU1* over-expression induces copper sensitivity in other media types (ie. YPD) is an open question which is beyond the scope of this work. We focused on a grape-juice like matrix because of this most closely reflects the environment in which these yeast are expected to operate.

Reviewer's Responses to Questions

**Comments to the Authors:**
**Please note here if the review is uploaded as an attachment.**

Reviewer #1: This manuscript demonstrates correctly the genetic interaction between SSU1 translocation and CUP1 copy number on copper tolerance. The molecular mechanisms behind this interaction are still to be elucidated. However, this manuscript rigourously reject many potential causes and shows a link with sulfur limitation.
This work is original and of importance for researchers in the field.

Before recommending publication I would like to have precisions regarding the following comments :


General major comments :

Reviewer: I am confused with these sentences :
L99 : "The diverse CUP1 copy number variation among strains in the wine yeast clade is consistent with the previously observed fitness variation in high copper medium [34]. However, there was a poor correlation between CUP1 copy number and relative strain fitness in a medium containing 10 mg/L copper (Fig 1)"

I'm not sure to what "consistent" is referring to, and which concentration of copper medium is considered as high. In ref [34], 10 mg/L copper seems to be also used. Therefore, I don't understand the contrast between the two sentences. I suggest to show the relationship between CUP1 copy number and strain fitness for the two level of copper. This will highlight at which concentration the trade-off is present.

Author response:

We agree with the reviewer that the sentence appears ambiguous. We have rewritten the sentence in an attempt to improve clarity as follows;

“Although there was a high diversity in both CUP1 copy number and the previously observed strain fitness in medium containing 10 mg/L copper [34], and a relationship between copy number and fitness was anticipated, no correlation between genetic and physiological characteristics was observed (Fig 1).”

Reviewer: Regarding the title of the paragraph
(L 92-93) High CUP1 copy number is necessary but not sufficient in determining copper tolerance in wine yeast
Fig 1 is not showing that as some strains appear to be resistant (266 or 1722) without having high CUP1 copy number. As mentioned in the subsequent text, in this condition no link between CUP1 and strain fitness is found.

Author response:

We have reworded the title to better reflect the contents of this section as follows;

“CUP 1 copy number alone is a poor predictor of copper tolerance in wine yeast”

Reviewer: The fact that no relation between CUP1 and strain fitness is found should be more discussed as many papers even cited in this manuscript demonstrated a link between CUP1 copy number and copper tolerance.
It may be specific to the condition tested (copper concentration) or to the genetic background (wine yeasts that are enriched in SSU1 translocation).

Author response: We are not entirely sure what the reviewer is asking of us with this comment. We are not claiming that CUP1 copy number is not important in the provision of copper tolerance. In fact, the QTL analysis of F1 progeny from a cross between Cu^tol^:CUP1^high^ x Cu^sen^:CUP1^low^ strains demonstrated that high CUP1 copy number is a key feature of copper tolerance in wine yeast. This finding is consistent with the work of others and demonstrates that the copper tolerance phenotypes being investigated here are not specific to the condition being tested.

The subsequent QTL analysis, of a cross between Cu^tol^:CUP1^high^ x Cu^sen^:CUP1^high^ demonstrates the contribution of genetic features not previously associated with copper tolerance (SSU1 translocation).

To clarify this section we have reworded the paragraph describing the QTL analysis of F1 progeny from a cross between Cu^tol^:CUP1^high^ x Cu^sen^:CUP1^low^ strains as follows;

“An analysis of the SNP frequency in F1 progeny from AWRI 3001 (Cu^tol^:CUP1^high^ x Cu^sen^:CUP1^low^) was undertaken. A clear divergence from the 1:1 expected parental genotypic ratios across the sensitive and resistant bulk pools was observed at base pair 210,000 on Chr VIII. This position corresponds almost exactly to the location of *CUP1-1* and *CUP1-2* (Fig 2A). The major association on Chr VIII was consistent with the mean difference in *CUP1* copy number between the two strains (Δ copy number = 23.1 copies [95CI, 19.9, 26.3]). This data explains the copper sensitivity of the AWRI 1537 strain and supports previous observations [18,19] that CUP1 is a key determinant of copper tolerance in yeast.”

Reviewer: 108 -109 : Poor predictor in this specific condition. As mentioned in the introduction the association is described in other papers.

Author response:

We agree that the association of CUP1 with copper tolerance has been described and don’t dispute this. As pointed out above, our QTL analyses show that the metallothionine CUP1 is still important for copper tolerance in wine yeast in the conditions in which we evaluated them. We would also point out that other authors have shown that Peter et al 2018 also pointed out that copy number expansion only explains 44.5% of phenotypic variation in copper tolerance across a wide selection of cerevisiae yeasts, not only wine yeast.

We have attempted to better reflect our position by slightly rewording this sentence as follows;

“The observation here and elsewhere [20] that CUP1 copy number is a poor predictor of copper tolerance in yeast raises the obvious question; why are strains with a high CUP1 copy number so poorly tolerant of copper in the medium?”

Reviewer: I have another major concern regarding the GO category as shown in Fig4 panel C.
It seems that many genes are not with correct GO category. For exemple *AUS1* is a sterol transporter and has not the sulfur compound transport GO:0072348 term. I suggest to check this issue and rerun the analysis if necessary.

Author response:

We thank the reviewer for commenting on the GO categories. Admittedly this has caused some internal discussion about the best way to present this information. As the reviewer would be aware, the hierarchical structure of GO ontology terms contains a high level of redundancy between terms. Several Over Representation Analysis (ORA) methods seek to reduce confounding data interpretation that can arise by extracting representative terms for the redundant categories. The analysis pipeline that we used was Metascape. Metascape computes Kappa similarities between all pairs of enriched terms and uses this to hierarchically cluster the terms to generate groups of similar terms. The GO categories that we used in the original submission are summary categories for those groups of terms that cluster together. We agree that these summary terms are misleading and don’t accurately represent the genes contained within them.

To resolve this issue we have rerun the Over Representation Analysis focusing on Biological Processes. The repeat analysis has now resolved the summary category ‘Sulfur Compound Transport’ into two summary categories: ‘Sulfur Compound Transport’ and ‘Transmembrane Transport’. The new summary category still contained 7 genes not directly related to sulfur compound transport however, these were also contained within other GO summary categories, specifically ‘Transmembrane Transport’. Therefore, for the ‘Sulfur Compound Transport’ group we have displayed only those genes that can be specifically associated with sulfur compound transport.

Other summary categories that displayed genes already contained within larger or more general summary terms were not included in the figure. These were ‘sulfate transport’ whose genes were contained within ‘sulfur compound transport’, and ‘fermentation’ whose genes were contained within ‘primary alcohol metabolic process’.

A revised figure 4 has now been provided that we believe provides a more accurate representation of differentially expressed genes. We also believe that this addresses the concerns of reviewer 1.

In addition, we have amended Line 185 with an updated Log(P) value for sulfur compound transport and included an amended S4 Fig in the supplementary file.

Minor specific comments

Reviewer: The introduction presents SO2 and copper resistance mechanisms. However, the potential interaction between them is presented in only two sentences L76 to L78. As it is the main subject of the manuscript I suggest to add more detail regarding the data supporting their interaction (ref 33,34) and hypothesis behind that.

Author response:

Ref 34 simply noted that copper and SO2 tolerance among wine yeast appeared to be mutually exclusive. The observation was made without any hypothesis as to its cause. That observation stimulated the work reported here directly.

Ref 33 suggests that upregulation of sulfur amino acid metabolism in high copper environments is likely due to a requirement for glutathione in oxidatively stressed cells, helping to control the inactivation of iron-sulfur cluster containing proteins.

We have provided more detail by adding the following sentences to the introduction with in text citations and reference list updated accordingly;

“Specifically, Hodgins-Davis et al. [33] show that both copper starvation and toxicity elicit responses from genes associated with sulfur metabolism. CUP1 poor strains were shown to respond to increased copper concentrations with upregulation of genes related to mitochondrial activity or oxidative stress. These cellular functions are also critical contributors to survival in multiple forms of starvation (Petti et al) and are part of a larger response to starvation which ultimately results in cell cycle arrest (Boer and Botstein).

In other work, Fay et al (ref) observed upregulation of sulfate assimilation pathway genes in a subset of strains during a study of copper stress but could not directly associate this as a response to copper, but rather with the capacity of those strains to become discolored through the formation of CuS. de Freitas et al (ref) showed that addition of copper could rescue the ability of Δssu1 yeast to grow on non-fermentable carbon sources. This was attributed to depletion of copper or iron by accumulation of intracellular sulfur compounds. Taken together these works provide a picture of the strong interdependence of copper and sulfur metabolism in yeast.”

Reviewer: S3 fig. L118 in the manuscript is mentioning AWRI796, AWRI 1537 and AWRI 1487, figure shows 3019, 3029 and 3032

Author response:

We thank the reviewer for picking this up. AWRI 3019, 3029 and 3032 are molecularly barcoded versions of AWRI 796, 1487 and 1537 respectively, and are the correct designations for them. The text has been changed as follows;

The parental copper-resistance phenotype was present in all spores isolated from AWRI 3019, AWRI 3029 and AWRI 3032 (S3 Fig), corresponding to barcoded versions of AWRI 796, AWRI 1487 and AWRI 1537, respectively.

Reviewer: Line 169: It would be easier to get the construction by mentioning directly that AWRI 4052 was generated from AWRI 3471. You can also add panel A in figure quote.

Author response;

We understand that following all the different names of these strains in the text is difficult and we have struggled with the least confusing but still accurate ways of presenting them. We therefore welcome suggestions about how to improve the readability of the manuscript. However, in this case the text does already specifically point out that “Except for the promoter of SSU1, AWRI 4052 is genetically identical to AWRI 3471.”

In addition, we have added the panel label to the in-text reference to the figure and added the following (shown in red) to the sentence;

“Fig 4A also shows the growth of AWRI 4052 (ssu1(p)Δ::pECM34(p)), in which the wild-type promoter of *SSU1* in the AWRI 3471 background has been exchanged for the ECM34 promoter from AWRI 1487.”

Reviewer: L187 : Fig4 shows that a considerable number of genes associated to sulfate / sulfur GO term have also decreased expression in AWRI 4052. The manuscript only highlight increased expression cases. Comments on other genes would be interesting.

Author response:

At least half of this section contains a discussion of genes that are down-regulated in this contrast, including commentary on genes related to thiamine metabolism and cell wall structure.

While we agree with the reviewer that there is a lot of information in this data set that is not specifically presented (ie. we have left out any discussion of comparisons of low and high copper conditions), we have deliberately attempted to be concise with this section to leave space for the presentation of information relating to understanding the causes of SSU1 suppression of copper tolerance in CUP1 carrying yeast.

Reviewer: Fig4 panel C. I suggest to mention SSU1 as a control. SSU1 is not part of any of these GO categories?

Author response:

SSU1 was excluded from the over-representation analysis (ORA) because its expression level is not a dependent variable. It was part of the experimental design and therefore including it in the ORA analysis would have had confounding effects on the outcome. To clarify we have added the following sentence to the methods;

“As an independent variable in the experiment SSU1 was omitted from the gene list submitted to the Metascape ORA.”

We have also added the following the legend of Fig 4 panel C;

“SSU1 was omitted from over representation analysis and therefore does not appear in enriched ontology summaries.”

Reviewer: Fig4 panel D. Caption is mentioning red and blue colors but it’s orange and blue. You may keep the same color code blue/red used in previous panels when comparing AWRI4052 - AWRI3471 in i) and ii) subpanels and choose a different in iii) iv).

Author response:

We have homogenized the colour palettes used in Figure 4 panel D i) and ii) with the rest of the figure as suggested and adopted a lighter hue for high and low contrasts of the same strain in panel D iii) and iv).

Reviewer: L246 and Fig4 panel D, subpanel ii). Figure would be easier to read with SSU1 labelled on the figure. According to the text in the manuscript it still have increased abundance.

Author response:

The adjusted P value for Ssu1p was below above the 0.005 cutoff score used to visualize this dataset and therefore was not shown by default. We have now included it as an additional annotation.

Reviewer: L413 Error!

Author response:

An appropriate citation has been inserted at this position.

Reviewer: SO2 concentration is indicated either in mg/L or in µM according to text or figure. Homogenization should be good

Author response:

We believe the reviewer is referring to SO4 concentrations. We have now changed the references in the text to mg/L except for the first instance on Line 348 where we have given both to facilitate comparison with literature values.

Reviewer: L320 S6 fig : I guess that x axis scale is wrong as the manuscript is describing a concentration of 20 mg/L (L320)

Author Response:

We thank the reviewer for picking this up. The scale was originally in g/L. This has now been amended read as to mg/L to be consistent with the rest of the manuscript.

Reviewer: L347 % of variance explained would be interesting

Author Response:

Apologies to the reviewer in this point but we do not understand what they are referring to with this comment.

Reviewer: Fig 7 is lacking the strain AWRI 3471 as control.

Author response:

We did not include AWRI 3471 in this experiment. We had already shown that AWRI 3471 is unaffected by 10 mg/L of copper when growing in medium containing limited SO4 (Fig S7). To highlight this point we have added the following sentence to the manuscript;

“In contrast, the growth of AWRI 3471 in SO4 limited medium was largely unaffected by 10 mg/L copper and sugar utilisation was only slightly delayed (S7 Fig).”

Reviewer #2: This manuscript is reporting a detailed analysis of the tradeoff between copper and SO2 tolerance in S. cerevisiae. The paper is significantly progressing our understanding of the molecular determinants responsible for the mutually exclusive tolerance to these 2 compounds. The work is rigorously done and very convincing. I have only minor suggestions to improve the quality of the paper.

Minor modifications:

INTRODUCTION:

Reviewer 2: - The introduction is pleasant to read and nicely introduces the general question on the genetic determinants controlling copper and SO2 tolerance in yeast. However, the authors should mention that in addition to translocations, an inversion on chrXVI was reported to also promote sulfite tolerance (PMID: 30859719). Furthermore, it would be worth mentioning that the COM2 regulon has also been involved in the tolerance to sulfur dioxide (PMID: 31799324).

Author response:

We thank the reviewer for these suggestions. We have now incorporated them into the introduction.

RESULTS:

Reviewer 2: - In the first paragraph the authors indicate that their qPCR estimates are “consistent” with the copy number estimated from whole-genome sequence data. However, there is ~2 fold difference between the 2 studies across the entire range of values that should at least be commented. In addition, the CUP1 copy number per strain should be carefully verified as there seems to be some inconsistencies between the y-axis of Fig1 and the y-axis of Supp Fig2, which are supposed to represent the same data. For instance, the strain 2914 has nearly 40 copies based on Schmidt Supp Fig 2 while it goes down to only about 32 on Fig 1. Finally, the presence of the barcode that is used to estimate the CUP1 copy numbers in this study should be better recalled than ‘described in [34]’ and it would be also nice to explain how the relative strain fitness was calculated.

Author response:

We thank the reviewer for picking up the discrepancies between Fig 1 and Sup Fig2. Sup Fig2 was prepared with an earlier version of the CNV data set. Both figures have now been prepared from the same curated data set. The names of the strains shown in both figures have also been altered to be consistent with the strain terminology used in the rest of the manuscript.

The reviewer is correct in their observation that there is a two-fold difference in the copy numbers of CUP1 reported here and those reported by Steenwyk et al 2017. The difference arises because of the difference in approaches used. The whole genome mapping approach used by Steenwyk will normalize against the average genome read depth of a diploid organism. The average read depth of a diploid organism mapped against the haploid S288c in Steenwyk is used as a proxy for copy number and is equated to a copy number of 1. But in polyploid organisms there are N copies each ‘single copy’ gene. Therefore, in the Steenwyk work a diploid wine yeast strain with two physical *CUP1* gene copies present will have a read depth when mapped to S288c equal to the average read depth and be reported as having a copy number of 1. We refer to this in the manuscript as haplotype copies. None of this is spelt out explicitly by Steenwyk but can be inferred from the approach that was taken by these authors.

With our analysis we normalized against a barcode inserted into the genome of the organism. That barcode is present as a single copy in one of the two copies of HO within the diploid wine yeast. Because we normalize against a molecular feature that we know is present as a single copy the number of copies that we estimate will be an absolute number of gene copies. We refer to it as such in the text. The absolute number of any gene in a diploid organism will always be twice the number of haploid copies hence the slope of the regression line in S2 Fig approaching 0.5.

Reviewer 2: In the second paragraph, we expect to see the growth of spores isolated from the AWRI 796, AWRI 1537 and AWRI 1487 strains but the numbers are different in Supp Fig 3 which makes the story very difficult to follow (3019, 3029 and 3032). The reader has to refer to Table 1 to follow the strain names. I t would be much better to keep a single name all across the main text and in all figures.

Author response:

A similar comment was made by Reviewer 1. We have changed the names of the strains in this paragraph to reflect the names in Supp Fig 3 as follows;

“The parental copper-resistance phenotype was present in all spores isolated from AWRI 3019, AWRI 3029 and AWRI 3032 (S3 Fig) corresponding to barcoded versions of AWRI 796, AWRI 1537 and AWRI 1487, respectively.”

Reviewer 2: In the third paragraph, for the QTL analysis of the AWRI3471, the authors report a minor QTL at ~1,000,000 bp that is very difficult to locate on Figure 2 because the genome position scale is not readable. In addition, there are many other deviations from the 0.5 expected proportion, in several chromosomes (including the entire chromosome I), that are disregarded without being at all discussed.

Author response:

The images in Figure 2 have been revised to include scale indicators.

The deviation from the expected 0.5 proportion for the entire chromosome 1 in AWRI 3471 (barcoded AWRI 796) observed by reviewer 2 is the result of a potential whole chromosome amplification of chromosome 1. This has been reported previously by Borneman et al (2011). This observation has been added to the text at the end of the relevant section as follows;

“A divergence from the expected 0.5 SNP ratios for the entirety of Chr I was observed in diploids derived from both crosses (AWRI 3001 and AWRI 3811) and copper tolerant and copper sensitive pools prepared from the respective F1 progeny (Fig 2A and B). The divergence from expected SNP ratios for Chr I can be explained by a whole chromosome duplication that has been reported previously [42] in the progenitor strain used for this work (AWRI 796).”

Finally, we have been more specific with regard to the locations of QTL features in figure 2 and address the concerns about the lack of comment about the other minor QTLs by expanding on the paragraph as follows;

“An analysis of the SNP frequency in F1 progeny from AWRI 3001 (Cu^tol^:CUP1^high^ x Cu^sen^:CUP1^low^) was undertaken. One hundred and one positions across the genome were identified that exhibited a SNP ratio greater than 0.85. Sixty-five of those were on Chr VIII with a peak between 210,000 and 218,00 bp. This position corresponds to the location of *CUP1-1* and *CUP1-2* (Fig 2A). The major association on Chr VIII was consistent with the mean difference in *CUP1* copy number between the two strains (Δ copy number = 23.1 copies [95CI, 19.9, 26.3]). This data explains the copper sensitivity of the AWRI 3029 strain and supports previous observations [11,12,17,18] that *CUP1* is a key determinant of copper tolerance in yeast.

Of the remaining genomic locations with SNP ratios greater than 0.85, eleven of them were on Chr IV between positions 1,163,450 and 1,163,515 bp. However, the limited breadth of the change in SNP frequency around this location and that this, and other locations with high SNP ratios, were not mirrored in Cu-tol and Cu-sens pools suggest that there is no association with the phenotype.”

Reviewer 2: In the last paragraph of the Results (Sulfur limitation increases copper sensitivity in SSU1 over expressing yeast), there might be an issue with the units as the main text is reporting a threshold concentration of SO4 of 20 mg/L while in the Sup Fig 6, this threshold appears to be at 0.02 mg/L. The SO4 concentration in the figure should possibly be expressed in g/L.

Author response:

We thank the reviewer for picking this up. The scale shown in Sup Fig 6 was incorrect and was originally presented in g/L. The graph has been remade to show mg/L to be consistent with the rest of the manuscript.

Reviewer 2: The authors should consider the possibility of including the Supp Fig 7 as a panel of the main Fig 7.

Author response:

We thank the reviewer for the suggestion. For simplicity we prefer to leave Fig 7 in the manuscript as is. There are already a lot of complicated figures and believe that combining different experiments into the same figure may complicate things further.

METHODS:

Reviewer: There are 2 Error messages “Reference source not found” that need to be fixed.

Author response:

These have been addressed in response to Reviewer 1 above.

Reviewer #3: In this manuscript, Onetto et al. investigate the negative correlation between Cu and SO2 tolerance in S. cerevisiæ. Through segregant QTL-analysis, they identified the genetic variance causing Cu-sensitivity to be at SSU1, which is previously linked with SO2 tolerance. They show that overexpression of SSU1 effect on Cu tolerance is not caused by a weakening of the molecular defence against Cu toxicity. Instead, they show that Cu-sensitivity phenotype is metabolically linked to SO2 limitation. This is an interesting study that shed new lights on the mutual exclusivity of the two important traits that are Cu and SO2 tolerance in yeast. The works are very well conducted, the analyses are exhaustive, and they made a thorough investigation of the SSU1 dependant Cu-sensitivity. Here are minor points I believe would strengthen the paper.

Reviewer 3: 1. I would recommend the authors to explicitly mention the form of copper used in the experiments so reader can fully appreciate the nature of the cell stress.

Author response:

A full description of the media has now been added into the methods and materials section (Yeast Strains and Culturing). The added description of the medium now reads;

“Experiments were performed in a defined medium [7], the composition of which resembles a Chardonnay juice. Briefly, the defined medium composition was consisted of (per litre): glucose 100 g, fructose 100 g, citric acid 0.2 g, malic acid 3 g, tartaric acid 2.5 g, K2HPO4 1.1 g, MgSO4 .7H2O 1.5 g, CaCl2 .2H2O 0.4 g, H3BO3 0.04 g, proline 0.84 g, nitrogen as ammonium and amino acids to 300 mg N/L of yeast assimilable nitrogen (YAN), trace elements stock solution 1 mL, vitamins solution 1 mL. Copper was added from a 40 g/L stock solution of CuSO4.5H2O to a 10 mg/L final concentration of copper ion unless otherwise indicated.”

Reviewer 3: 2. Figures would benefit, in terms of readability, from more detailed annotations of the genotypes. Especially in Fig4 A and B, a small space could be allowed to specify the mutation of SSU1 promotor.

Author response:

As mentioned above, we welcome suggestions to improve manuscript comprehension and thank the reviewer for their suggestion. We have now included the SSU1 genotype underneath the strain annotation in Figure 4. To increase the prominence, we have also moved the legend to the top of the Figure.

Reviewer 3: 3. In results section, line 173. The authors make the first mention about monitoring fermentation progress. I feel like the rational and explanations for this experiment should be stated more clearly (and the comment also apply to later in the results, line 328, at other mention of fermentation monitoring). Moreover, the implications of this results (delay in fermentation) for the following experiments is lacking.

Author response:

We agree with the reviewer comment. The measurement of fermentation progress does seem to come out of nowhere. To provide some context we have altered the text in this section and included a discussion of it in a new paragraph as follows;

“Fig 4B compares the fermentation performance of AWRI 3471 and AWRI 4052. In addition to the effects on growth, elevated copper concentrations in grape juice have been shown to impede sugar utilisation [43,44], an effect that is particularly relevant to commercial winemaking. Elevated copper concentrations effected fermentation progress in both AWRI 3471 and AWRI 4052, however, a severe delay in fermentation onset and significantly higher residual sugar concentrations were observed in fermentations by strain AWRI 4052 in copper excess relative to strain AWRI 3471 (mean increase of 78 g/L [95CI, 67, 86]) at day 17.

Reviewer 3: 4. Line 318, the authors refer to S6 fig as an initial screen that suggest a threshold concentration of 20mg/L SO4. However, the S6 fig shows almost unaltered growth for SO4 concentrations ranging 0.03 to 0.2mg/L. Maybe is it due to a mistake in S6 Fig labelling? Or other results are needing to fully support the threshold value?

Author response:

We thank the reviewer for picking this up. This was also noted by reviewer 1. The scale shown in Sup Fig 6 was incorrect and was originally presented in g/L. The graph has been remade to show mg/L to be consistent with the rest of the manuscript. It now shows growth to be unaltered for SO_4_ concentrations ranging from 30 to 200 mg/L.

Reviewer 3: 5. One topic I feel is not developed enough in the manuscript is the SO4 tolerance. Although the demonstration of Cu-sensitivity caused by SSU1 overexpression is strong, it let me wonder whether the system used (swap of endogenous SSU1 promoter for ECM34 promotor) is able to increase SO4 tolerance. I am not sure if such an assay is feasible. But testing SO4 tolerance in AWRI 4052 in comparison to AWRI 3471 would be a great characterization of the strain to better link Cu- and SO4-tolerance traits. If this was already tested in previous work, explicitly mention it would be recommended.

Author response:

This is a great suggestion, however, we have not tested SO_4_ tolerance and therefore cannot provide data for it. We have only tested strain response to SO_4_ limitation. We have given the possibility some thought and cannot think of a way to introduce SO_4_ into the medium without introducing a counter ion that also has a physiological effect.
